# DNA Extraction and Host Depletion Methods Significantly Impact and Potentially Bias Bacterial Detection in a Biological Fluid

**DOI:** 10.1128/mSystems.00619-21

**Published:** 2021-06-15

**Authors:** Erika Ganda, Kristen L. Beck, Niina Haiminen, Justin D. Silverman, Ban Kawas, Brittany D. Cronk, Renee R. Anderson, Laura B. Goodman, Martin Wiedmann

**Affiliations:** a Department of Food Science, Cornell University, Ithaca, New York, USA; b Consortium for Sequencing the Food Supply Chain, San Jose, California, USA; c IBM Almaden Research Center, San Jose, California, USA; d IBM T.J. Watson Research Center, Yorktown Heights, New York, USA; e College of Information Sciences and Technology, Penn State University, University Park, Pennsylvania, USA; f Department of Statistics, Penn State University, University Park, Pennsylvania, USA; g Department of Medicine, Penn State College of Medicine, Hershey, Pennsylvania, USA; h Department of Population Medicine and Diagnostic Sciences, College of Veterinary Medicine, Cornell University, Ithaca, New York, USA; Institute for Systems Biology

**Keywords:** host depletion, shotgun metagenomics, milk, DNA, RNA, biases, propidium monoazide, dairy, low biomass, food microbiome

## Abstract

Untargeted sequencing of nucleic acids present in food can inform the detection of food safety and origin, as well as product tampering and mislabeling issues. The application of such technologies to food analysis may reveal valuable insights that are simply unobtainable by targeted testing, leading to the efforts of applying such technologies in the food industry. However, before these approaches can be applied, it is imperative to verify that the most appropriate methods are used at every step of the process: gathering of primary material, laboratory methods, data analysis, and interpretation. The focus of this study is on gathering the primary material, in this case, DNA. We used bovine milk as a model to (i) evaluate commercially available kits for their ability to extract nucleic acids from inoculated bovine milk, (ii) evaluate host DNA depletion methods for use with milk, and (iii) develop and evaluate a selective lysis-propidium monoazide (PMA)-based protocol for host DNA depletion in milk. Our results suggest that magnetically based nucleic acid extraction methods are best for nucleic acid isolation of bovine milk. Removal of host DNA remains a challenge for untargeted sequencing of milk, highlighting the finding that the individual matrix characteristics should always be considered in food testing. Some reported methods introduce bias against specific types of microbes, which may be particularly problematic in food safety, where the detection of Gram-negative pathogens and hygiene indicators is essential. Continuous efforts are needed to develop and validate new approaches for untargeted metagenomics in samples with large amounts of DNA from a single host.

**IMPORTANCE** Tracking the bacterial communities present in our food has the potential to inform food safety and product origin. To do so, the entire genetic material present in a sample is extracted using chemical methods or commercially available kits and sequenced using next-generation platforms to provide a snapshot of the microbial composition. Because the genetic material of higher organisms present in food (e.g., cow in milk or beef, wheat in flour) is around 1,000 times larger than the bacterial content, challenges exist in gathering the information of interest. Additionally, specific bacterial characteristics can make them easier or harder to detect, adding another layer of complexity to this issue. In this study, we demonstrate the impact of using different methods for the ability to detect specific bacteria and highlight the need to ensure that the most appropriate methods are being used for each particular sample.

## INTRODUCTION

In the past decade, recent developments in molecular methods, including high-throughput sequencing (HTS) technologies, have demonstrated the feasibility of sequencing-based analysis of various foods and food-associated environments and the potential for application in informing food safety practices (1), product processing methods (2), and ingredient authentication (3, 4). This has spurred the efforts to translate the use of such technologies to the industry setting, with the objective of moving food safety testing to the next frontier (5). However, before such refined approaches can be reliably applied in the industry, it is imperative that appropriate methods be employed at every step of the process: gathering of primary material, laboratory methods, data analysis, and interpretation. In this study, we highlight the importance of considering the food matrix characteristics as well as the laboratory methodologies applied. Bovine milk is used as a model to demonstrate the several challenges associated with developing HTS methods for use in food matrices.

Like many other foods, milk is a chemically complex biological fluid. Milk contains several compounds that can hamper the chemistry involved in DNA and RNA extraction (6, 7) and act as PCR inhibitors, such as calcium ions, fats, and proteins (8, 9). Additionally, lactoferrin, an enzyme present in bovine milk, has recently been described to have both DNase and RNase activity (10). Another challenge for untargeted sequencing applications of bovine milk is the presence of bovine somatic cells. The bovine genome is 1,000 times larger than an average bacterial genome (bovine, 2.7 Gb; bacteria, 3.6 Mb [average], 3.4 Mb [median] [11, 12]). Thus, even when present in much smaller amounts than bacterial cells, bovine somatic cells introduce an enormous quantity of typically unwanted host nucleic acids in untargeted HTS studies. Realistically, however, high-quality raw milk may contain around 200,000 bovine somatic cells and 20,000 or fewer bacterial cells per ml (13), leading to a 10,000-fold higher abundance of bovine than bacterial DNA in high-quality raw milk.

Despite the challenges associated with nucleic acid extraction from milk and the amount of host DNA present, several investigations have successfully used milk (14–16) or other dairy products (17) as their sample of interest in targeted and untargeted HTS studies, highlighting the potential for application of HTS technologies in food production settings. Total RNA sequencing can be more informative than untargeted DNA sequencing, as it has the potential to provide gene expression (e.g., toxin production) in addition to taxonomic relative abundance in a community of food-associated microorganisms (4, 18). Compared to DNA extraction, RNA extraction is a more complex and challenging process, given the short half-life of RNA compared to that of DNA, its inherent susceptibility to degradation, and the known presence of RNases in milk (10). Nevertheless, a number of studies have been successful in extracting RNA from dairy products, highlighting the potential of RNA-based techniques for food safety and quality surveillance (19–21).

When the ultimate objective is to use HTS to create a tool that can be used to inform food safety and quality in industry settings, understanding the impact and biases introduced by using protocols that have not yet been tested or optimized for a given food matrix is of great importance. In addition, to be potentially adopted in industry settings, laboratory protocols should be performed in a timely manner. It is important to characterize the entire food sample, and most protocols available for nucleic acid extraction in milk begin with centrifugation and fat removal steps, which can in themselves introduce significant bias to the final result. For example, bacterial spores have been described to aggregate in the fat layer of milk samples subjected to gravity separation (22, 23), and RNA yields have been shown to vary among different milk fractions (24). Unfortunately, these potential introductions of bias are overlooked in most investigations.

While a number of studies have investigated commercially available protocols for nucleic acid extraction of milk in terms of DNA concentration, quality, and ability to detect specific pathogens (25–27), their main objective was not the assessment of differential DNA extraction or biases in the representation of diverse bacterial populations, which would require the inclusion of mock bacterial communities of interest in a milk sample and comparison of several protocols.

Host DNA contamination is a challenge not exclusive to milk and other food samples, as mammalian DNA has been shown to dominate the number of sequencing reads in cerebrospinal fluid, skin, vaginal, and oral metagenomes in humans (28–30). To tackle the host DNA issue, enzymatic and immunomagnetic protocols aimed at decreasing host DNA contamination became commercially available and have been tested in select sample types (28, 29, 31). In addition, a number of “homebrew” methods have been tested to allow for successful depletion of host DNA (28, 32, 33). Nevertheless, these methods are not guaranteed to work with every sample type, and to the best of our knowledge, no host DNA depletion methods have been evaluated for their applicability in milk.

The goals of this study were (i) to evaluate commercially available kits for their ability to extract nucleic acids (e.g., DNA and RNA) from bovine milk, (ii) to evaluate host DNA depletion methods for use with bovine milk, and (iii) to develop and evaluate a selective lysis-propidium monoazide (PMA)-based protocol for host DNA depletion in milk. Our overarching hypothesis was that methodologies would differ with regard to the efficacy of nucleic acid extraction, and potential biases would be observed. Experiments were thus performed on raw bovine milk inoculated with mock bacterial communities, which included Gram-negative (Salmonella enterica), Gram-positive (Listeria monocytogenes), and mycobacterial (Mycobacterium smegmatis) organisms, as well as spores representing aerobic sporeformers (Bacillus wiedmannii).

## RESULTS

### The extraction method significantly impacts bacterial quantification through qPCR.

**FIG 1:**
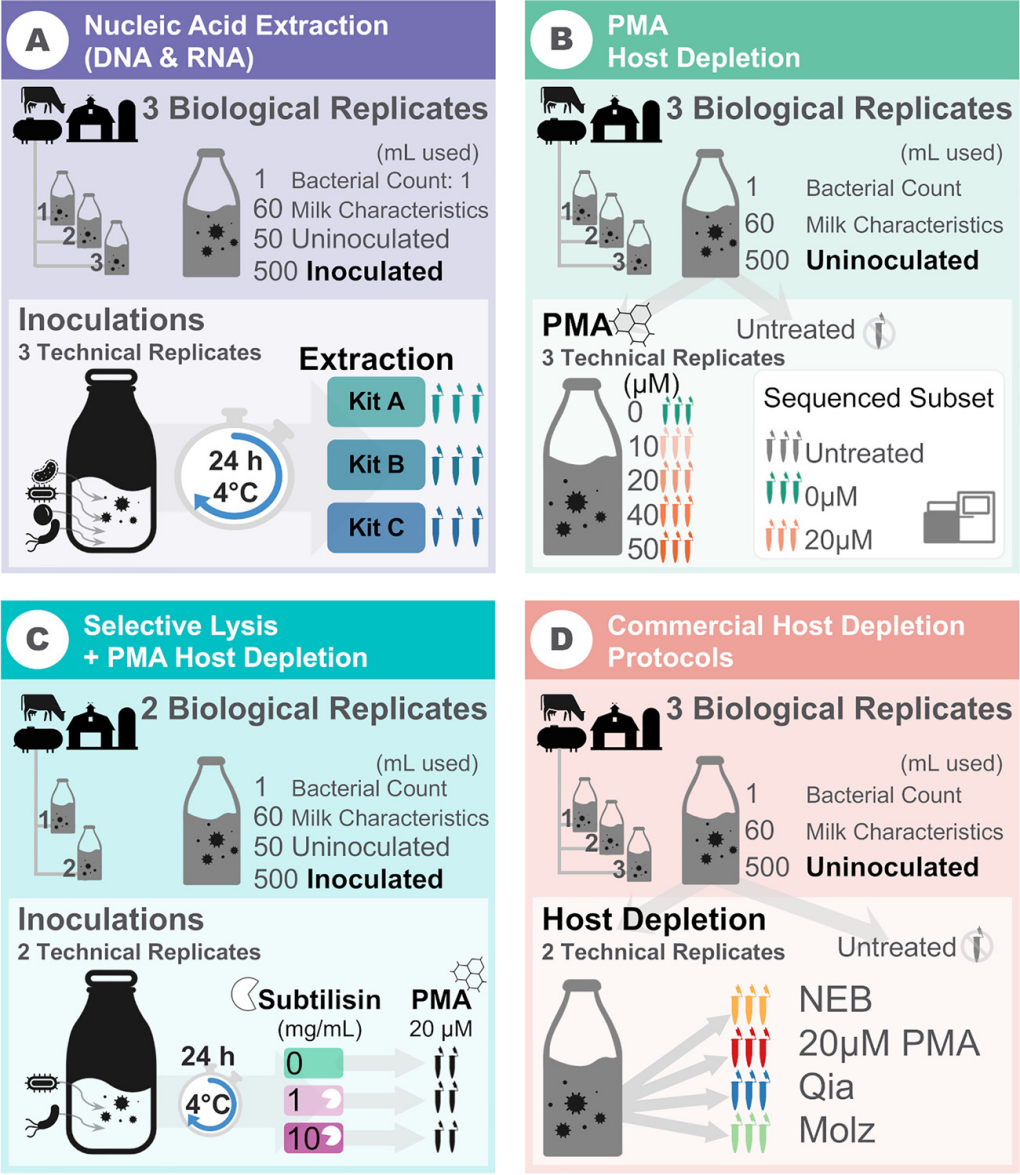
Study overview. Each panel depicts the design for each aspect evaluated, with respective numbers of biological and technical replicates. The qPCR assays were performed in duplicate for each experimental replicate. Copy numbers were calculated for bovine DNA, total 16S rRNA genes, *Bacillus*, *Listeria*, Mycobacterium, and Salmonella (A); bovine DNA, total 16S rRNA genes, *Listeria*, and Salmonella (C); and only bovine DNA and total 16S rRNA genes (B and D) because no inoculation was performed.

We assessed seven commercially available DNA extraction methods for their ability to isolate DNA from milk samples inoculated with a mock bacterial community (Fig. 1A) (for milk sample characteristics, see Table S1 in the supplemental material). Nucleic acid quantification and quality measurements (Table S2) need to be interpreted with caution due to the low overall DNA yield (<10 ng/μl for all samples), which placed readings below the linear range for most samples as measured via fluorescence with a Nanodrop. We were able to measure DNA for all kits via fluorescence with a high-sensitivity set of reagents, and all kits yielded sufficient DNA to allow for quantitative PCR (qPCR)-based quantification of the members of the bacterial mock community (i.e., Bacillus wiedmannii, Listeria monocytogenes, Mycobacterium smegmatis, and Salmonella sp.) as well as total bacterial 16S rRNA gene copies and bovine DNA.

10.1128/mSystems.00619-21.1 TABLE S1 Milk sample characteristics. Download Table S1, DOCX file, 0.03 MB. Copyright © 2021 Ganda et al. 2021 Ganda et al. https://creativecommons.org/licenses/by/4.0/ This content is distributed under the terms of the Creative Commons Attribution 4.0 International license.

10.1128/mSystems.00619-21.2 TABLE S2 Descriptive statistics of nucleic acid quantification and quality measurements. Download Table S2, DOCX file, 0.04 MB. Copyright © 2021 Ganda et al. 2021 Ganda et al. https://creativecommons.org/licenses/by/4.0/ This content is distributed under the terms of the Creative Commons Attribution 4.0 International license.

**FIG 2:**
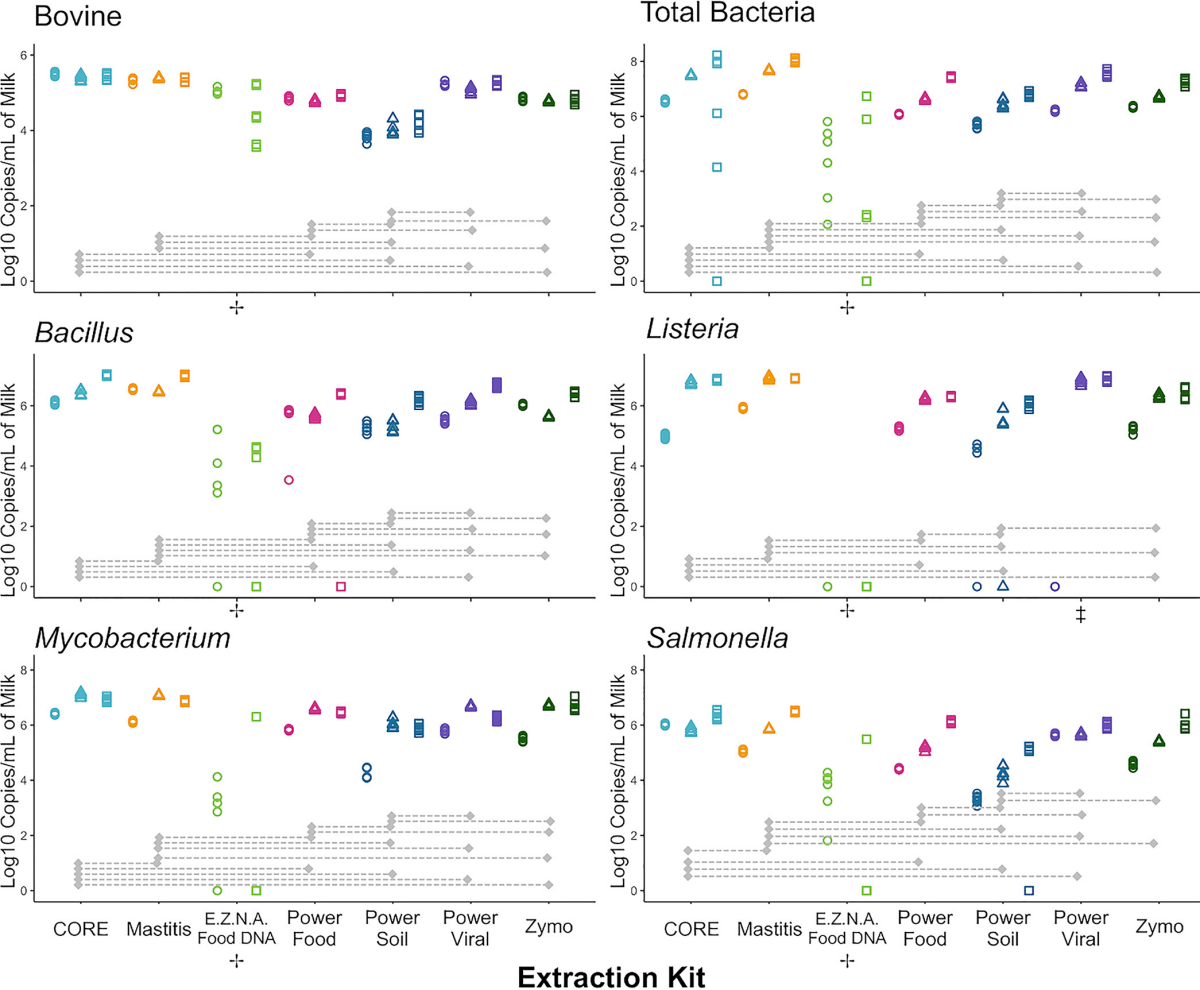
Scatterplots of normalized log copy numbers per milliliter of milk obtained with different DNA extraction kits. Points at 0 log copy numbers are graphical demonstrations of failed reactions. Gray bars represent pairwise significant differences at the 0.05 level after Bonferroni multiple-comparison adjustment within and between linear models, which included extraction kit, biological replicate, and their interactions. Scatterplot shapes represent each of three independent biological replicates (circles, first; triangles, second; and squares, third), whereas colors represent kits evaluated. Each panel depicts results of a single gene target evaluated. +, E.Z.N.A. Food DNA was not included in the second biological replicate and was excluded from all linear models; ‡ PowerViral was not included in the linear model for *Listeria*.

The failure rates varied across kits (Table 1; Fig. 2), with the MagAttract mastitis kit having no failed reactions and the MagMAX CORE nucleic acid purification kit having only one failed reaction in the total-bacteria assay. From the column-based methods, the E.Z.N.A Food DNA (EZFood) kit had the largest number of failed reactions, with a total of 30 failed reactions across five assays (all except for bovine DNA); because of this, we could not include data from this method in any of our statistical models, as this would have caused nonidentifiability. The Power Soil Pro (PSoilP) kit had 5 failed reactions in the *Listeria* and Salmonella assays, the Power Food (PFood) kit had only one failed reaction in the *Bacillus* assay, and the ZymoBIOMICS DNA/RNA (ZymoDNA or ZymoRNA) minikit had no failures. Interestingly, the AllPrep PowerViral DNA/RNA (PowerViralDNA or PowerViralRNA) kit had all reactions in the first biological replicate fail for the *Listeria* assay. As a result, we could not compare the PowerViral method to other methods in the *Listeria* assay.

**TABLE 1:** Descriptive statistics of log numbers of copies per milliliter of milk for DNA extraction

Organism	Mean log_10_ no. of copies per ml of milk as detected via qPCR (SD), no. of expts, ^no. of failures^						
	MagAttract mastitis	COREDNA	EZFood	PFood	PSoilP	PviralDNA	ZymoDNA
Total bacterial DNA	7.51 (0.54),18, ^0^	6.99 (0.96),18, ^1^	4.30 (1.72),12, ^2^	6.71 (0.57),18, ^0^	6.31 (0.48),18, ^0^	6.96 (0.59),18, ^0^	6.78 (0.40),18, ^0^
Bacillus wiedmannii	6.67 (0.25),18, ^0^	6.53 (0.39),18, ^0^	4.31 (0.77),12, ^4^	5.79 (0.67),18, ^1^	5.61 (0.45),18, ^0^	6.09 (0.49),18, ^0^	6.02 (0.32),18, ^0^
Listeria monocytogenes	6.58 (0.48),18, ^0^	6.21 (0.89),18, ^0^	NA (NA)^*a*^,12, ^12^	5.93 (0.49),18, ^0^	5.46 (0.68),18, ^3^	6.84 (0.09),18, ^6^	6.00 (0.59),18, ^0^
Mycobacterium smegmatis	6.70 (0.42),18, ^0^	6.81 (0.31),18, ^0^	3.97 (1.39),12, ^7^	6.29 (0.34),18, ^0^	5.44 (0.82),18, ^0^	6.25 (0.38),18, ^0^	6.30 (0.58),18, ^0^
Salmonella sp.	5.80 (0.61),18, ^0^	6.07 (0.26),18, ^0^	3.83 (1.12),12, ^5^	5.24 (0.72),18, ^0^	4.13 (0.77),18, ^2^	5.77 (0.19),18, ^0^	5.34 (0.61),18, ^0^
Bovine DNA	5.37 (0.05),18, ^0^	5.45 (0.08),18, ^0^	4.71 (0.59),12, ^0^	4.86 (0.08),18, ^0^	4.05 (0.24),18, ^0^	5.20 (0.11),18, ^0^	4.81 (0.06),18, ^0^

a NA, not applicable (this method did not yield detectable signal in qPCR for Listeria monocytogenes).

Of reactions that did not fail, the two magnet-based DNA extraction methods (the CORE and MagAttract mastitis kits, the two leftmost methods in Fig. 2) always yielded numerically higher log copy numbers (for all members of the mock community and for total bacterial 16S rRNA genes) than the other five kits. Bacterial copy numbers for the two magnet-based kits also had lower variability between technical replicates than column-based kits, with the E.Z.N.A Food DNA kit showing particularly large variability between technical replicates; DNA yields for this kit were also so low that no qPCR amplification was observed in the L. monocytogenes qPCR targeting *rpoB*.

Among the column-based extraction methods, Power Food, PowerViral, and Zymo were comparable with regard to (i) their ability to recover DNA of the members of the mock community and (ii) variability within technical replicates (Fig. 2; Table 1 provides detailed descriptive statistics). The Power Soil Pro kit (another column-based kit) generally provided lower bacterial DNA yields for each member of the mock community than these three kits.

We also evaluated the ability to isolate bacterial RNA for (i) two kits designed for isolation of RNA only and for (ii) three kits designed for isolation of both RNA and DNA (Table 2). As with the DNA detection data detailed above, all kits yielded very low total nucleic acid concentrations based on spectrophotometry. Reverse transcriptase qPCR (RT-qPCR) amplification of gene targets for Salmonella sp. and L. monocytogenes as well as bovine RNA did not yield amplification from samples treated with DNase (these initial tests were performed on two technical replicates for each of the four kits). Control amplifications on nucleic acids before DNase treatment, however, yielded amplification with cycle threshold (CT) values that did not differ between the RT-qPCR and the control qPCR targeting DNA, suggesting the presence of residual DNA in the extracted nucleic acids and demonstrating that, by following the protocols described here, we were not able to successfully isolate RNA from our milk samples.

**TABLE 2:** Nucleic acid extraction kit characteristics

Abbreviation(s)	Kit	Sample input (μl)	Nucleic acid output (μl)	Nucleic acid	Processing	Capture	Catalog no.	Manufacturer
PowerViralDNA or PowerViralRNA	AllPrep PowerViral DNA/RNA kit	200	100	DNA/RNA	Manual	Column based	28000-50	Qiagen
COREDNA, CORERNA	MagMAX CORE nucleic acid isolation kit	200	90	DNA/RNA	Automated	Magnet based	A32700	ThermoFisher
ZymoDNA, ZymoRNA	ZymoBIOMICS DNA/RNA minikit	250	100	DNA/RNA	Manual	Column based	R2002	Zymo
Mastitis	MagAttract mastitis kit	400	100	DNA	Automated	Magnet based	947757	Qiagen
PFood	DNeasy PowerFood microbial kit	1800	100	DNA	Manual	Column based	21000-100	Qiagen
PSoilP	PowerSoil Pro kit	500	100	DNA	Manual	Column based	47014	Qiagen
EZFood	E.Z.N.A. Food DNA kit	500	100	DNA	Manual	Column based	D4616-00	Omega
RNeasy	RNeasy Protect Bacteria minikit	1,500	50	RNA	Manual	Column based	74524	Qiagen
EZNARNA	E.Z.N.A. HP total RNA kit	500	50	RNA	Manual	Column based	R6812-00	Omega

### Osmotic lysis followed by PMA treatment decreases host DNA but also impacts bacterial DNA.

**FIG 3:**
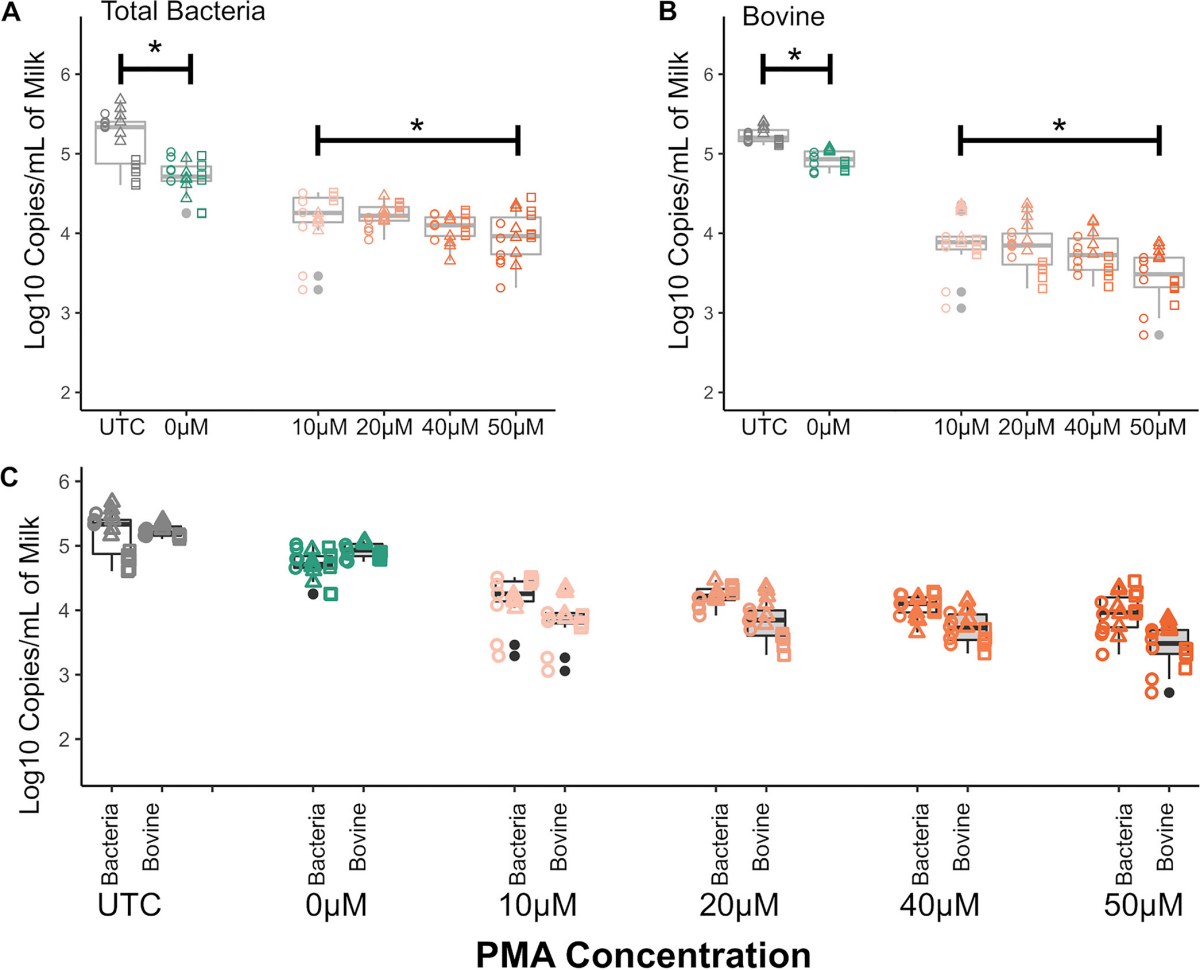
Effect of osmotic lysis of raw milk followed by treatment with various PMA concentrations on host and bacterial DNA counts as determined by qPCR. Boxplots represent normalized log copy numbers per milliliter of milk. UTC, untreated control sample; 0 μM, milk that underwent osmotic lysis but was not treated with PMA. Scatterplot shapes represent each of three independent biological replicates (circles, first; triangles, second; and squares, third), whereas colors represent concentrations evaluated. Asterisks represent significant differences at a *P* of <0.05 in linear-model comparisons. Osmotic lysis significantly decreases log_10_ copy numbers in non-PMA-treated samples (UTC versus 0 μM), and increasing PMA concentrations significantly decrease log_10_ copy numbers in a dose-dependent manner.

We compared protocols that combined osmotic lysis and treatment with multiple concentrations of PMA for their ability to reduce host DNA, compared to bacterial DNA copy numbers. To assess whether osmotic lysis alone impacted copy numbers, we compared untreated control (UTC) samples to samples that underwent centrifugation and osmotic lysis with double-distilled water (ddH_2_O) but without PMA addition (0 μM PMA); this comparison revealed significantly lower bovine and bacterial DNA copy numbers in 0 μM PMA samples, suggesting significant effects of sample processing (including lysis) independent of PMA addition (Fig. 3, UTC versus 0 μM comparisons). We also observed that PMA concentration significantly impacted bovine and bacterial DNA copy numbers in a dose-dependent manner; as the PMA concentration increased, a sharp decrease was observed in bovine copy numbers, whereas a less pronounced, but still noticeable, decrease was observed in bacterial copy numbers (Fig. 3). This trend was confirmed in the model estimates, which showed a significant and negative effect of PMA in both linear models, with a greater estimate for bovine copy numbers (−0.022) than for bacterial copy numbers (−0.012). Treatment with 20 μM PMA was the concentration deemed optimal, as it yielded the greatest decrease in host DNA without critically compromising bacterial DNA recovery and thus was chosen for subsequent comparisons with commercially available host DNA depletion protocols.

We also selected a small subset of milk samples from each of the three biological replicates to undergo deep untargeted sequencing, including one sample each of the three biological replicates representing (i) untreated samples, (ii) samples treated with 20 μM PMA, and (iii) samples that underwent osmotic lysis but were not treated with PMA (they underwent centrifugation and selective lysis with ddH_2_O but were not treated with PMA), which were included to allow us to determine if differences observed would be due to PMA treatment or sample processing prior to PMA addition.

**FIG 4:**
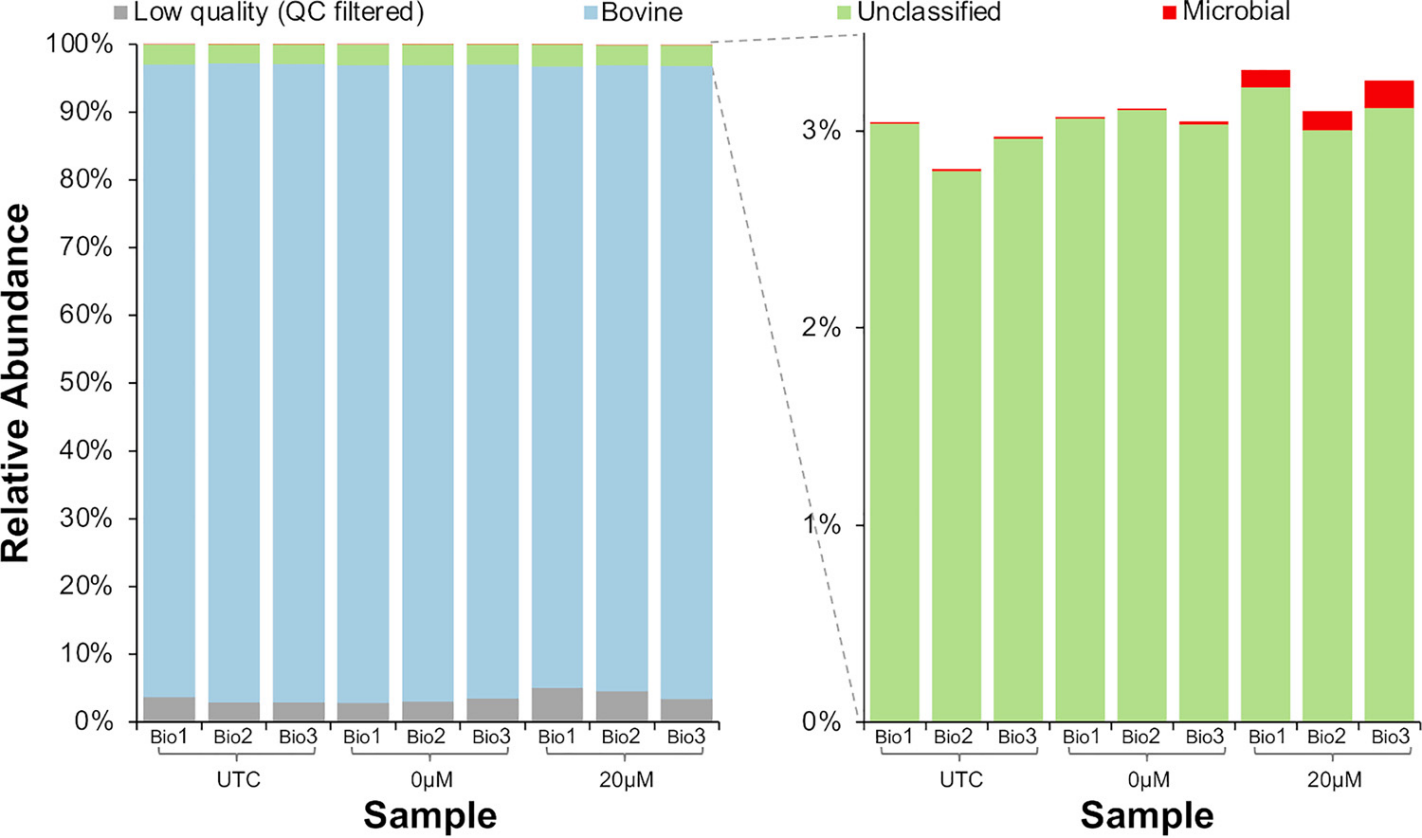
Effect of host DNA depletion with PMA on the percentage of sequencing reads assigned to the bovine genome or microbial genomes. Raw milk was collected on three separate days and divided into aliquots that underwent each of the processing methods. UTC, untreated control sample; 0 μM, milk that underwent osmotic lysis but was not treated with PMA; 20 μM, milk that underwent osmotic lysis and PMA treatment at 20 μM.

Sequencing had an average read depth of 51,563,707 reads per sample (range, 25,488,728 to 127,203,413 reads). Overall, less than 1% of all reads that passed quality control (QC) were determined to be of microbial origin regardless of PMA treatment (Table 3; Fig. 4), and around 3% remained unclassified.

**TABLE 3:** Descriptive statistics of shotgun metagenomics sequencing results assessing the effect of PMA treatment

Parameter^*a*^	Mean % (SD), no. of expts^*b*^		
	No treatment	0 μM PMA (wash only)	20 μM PMA
Bovine	93.87 (0.54), 3	93.83 (0.366), 3	92.46 (0.866), 3
Microbial	0.010 (0.002), 3	0.011 (0.005), 3	0.109 (0.027), 3
Unclassified	2.933 (0.122), 3	3.066 (0.04), 3	3.113 (0.11), 3
Low quality (QC filtered)	3.18 (0.441), 3	3.09 (0.384), 3	4.31 (0.851), 3

a Indicates to which class (e.g., eukaryotic, bacterial, viral, archaeal) sequencing reads were assigned using methods described in the work of Beck et al. (4).

b The average numbers of input reads were 67,983,431, 45,846,439, and 40,861 for the no-treatment, 0 μM PMA, and 20 μM PMA experiments, respectively.

**FIG 5:**
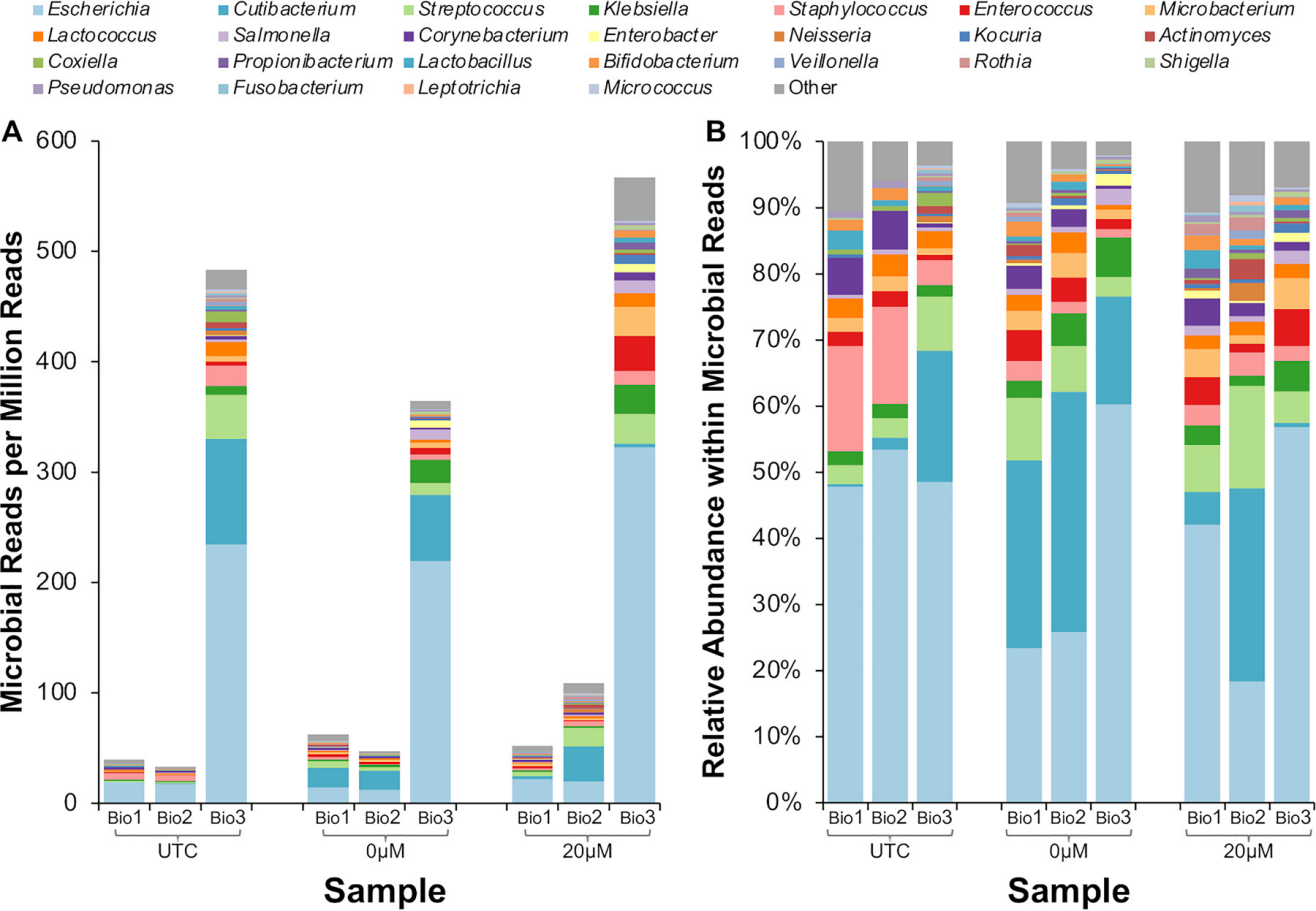
Microbial profile of samples exposed to osmotic lysis and PMA treatment. (A) Microbial reads per million reads by biological sample and treatment. (B) Relative abundance within microbial reads by biological sample and treatment. UTC, untreated control sample; 0 μM, milk that underwent osmotic lysis but was not treated with PMA; 20 μM, milk that underwent osmotic lysis and PMA treatment at 20 μM.

While we observed a pronounced increase in the number of bacterial reads per 1 million sequenced reads with the PMA treatment (Fig. 5A), attempting to deplete host DNA with PMA also appeared to influence the resulting microbial profiles of samples, leading to visible differences between replicates of the same biological samples (Fig. 5B). For example, PMA treatment of sample Bio1 resulted in greater relative abundances of *Cutibacterium* organisms than occurred in aliquots of the same sample (Bio1) that were not treated (UTC) or underwent selective lysis with ddH_2_O but received no PMA treatment (0 μM). Similar differences between the UTC and the 0 μM and 20 μM PMA samples were observed for the other two samples sequenced (Bio2 and Bio3) (Fig. 5B).

### Host depletion methods successfully decrease host DNA but not to the extent needed to make a significant impact for use in milk sequencing studies.

**FIG 6:**
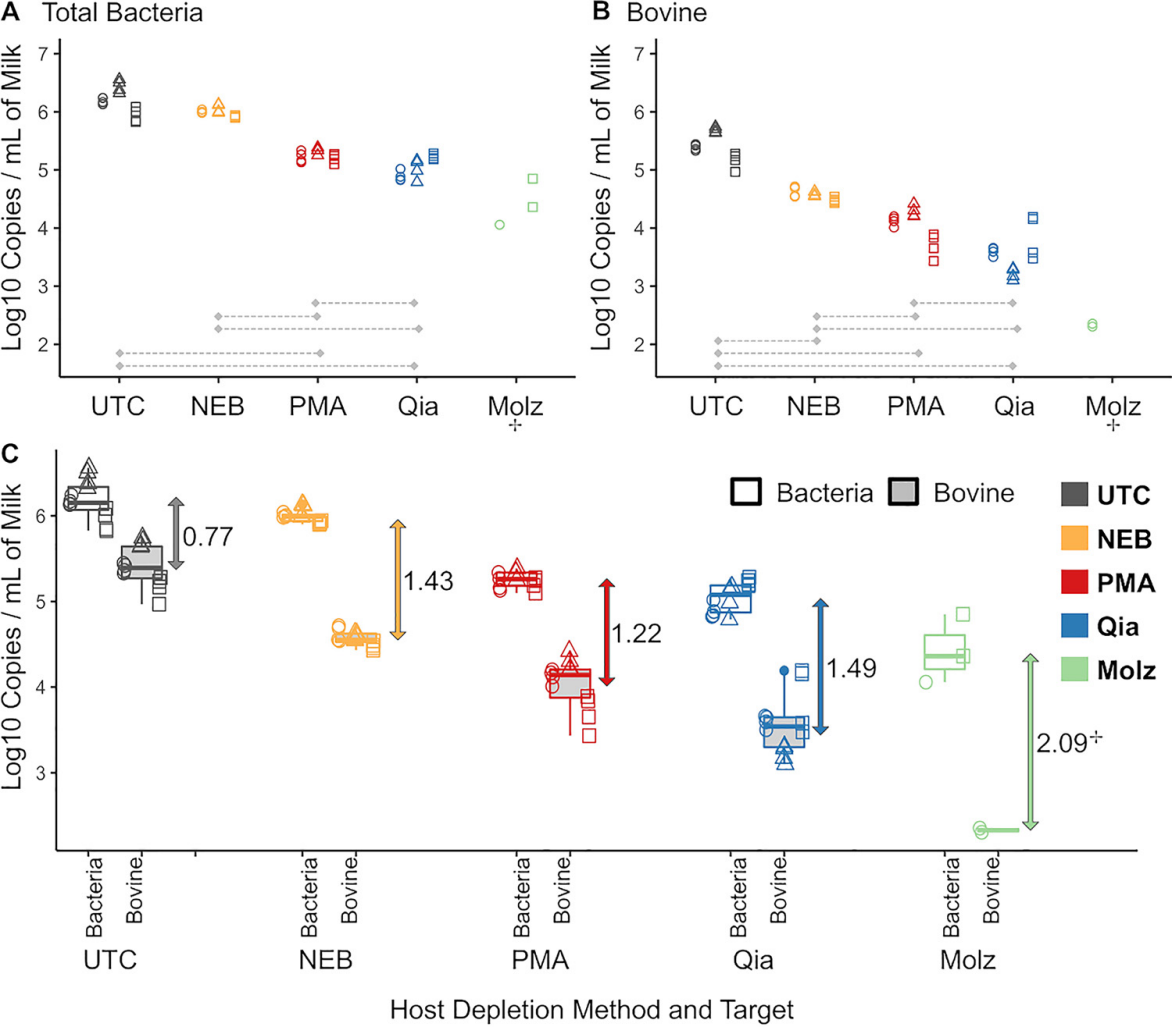
Comparisons of host depletion methods in uninoculated milk. (A and B) Scatterplots of normalized log copy numbers per milliliter of milk obtained with different host depletion methods for total bacterial copy numbers (A) and bovine DNA copy numbers (B). Gray bars represent pairwise significant differences at the 0.05 level after Bonferroni multiple-comparison adjustment in a linear model that included the host depletion method, biological replicate, and their interaction. Scatterplot shapes represent each of three independent biological replicates (circles, first; triangles, second; and squares, third), whereas colors represent methods evaluated. (C) Paired boxplots represent normalized log copy numbers per milliliter of milk from three independent experiments with two technical replicates each, except with the Molzym experiment, which had technical replicates performed on only 1 day. Numbers represent the log difference between mean log bacterial copy numbers and mean log bovine DNA copy numbers. +, technical replicates were performed on only 1 day due to the sample becoming an insoluble pellet during processing; therefore, this method was not included in comparisons. Qia, Qiagen; Molz, Molzym.

We compared the results of host DNA depletion methods and osmotic lysis followed by PMA treatment in three independent biological replicates. In each biological replicate, a freshly collected milk sample was homogenized and divided into five separate aliquots that were processed in parallel for each of the five methods (e.g., PMA method, Molzym [Molz] kit, NEBNext microbiome DNA enrichment [NEB] kit, QIAamp DNA microbiome [Qia] kit, and untreated control [UTC]). The means by which host DNA depletion was accomplished varied between methods; some kits depended on host DNA degradation followed by treatment with different enzymes (e.g., Qiagen and Molzym kits), while the NEB kit was based on the capture of methylated host DNA (for details on kits and methods, see Table 4; for descriptive statistics, see Table 5). Overall, host DNA removal treatments significantly decreased bovine copy numbers compared to those of an untreated control (Fig. 6B) (*P* < 0.001). However, we also observed a significant loss of bacterial copy numbers across methods (Fig. 6A) (*P* < 0.001). Notably, the Molzym kit caused the largest reduction of both bovine and bacterial DNA copy numbers, to levels that likely would be insufficient as inputs for shotgun metagenomic sequencing. Treatment of raw milk with Molzym reagents specifically resulted in a pellet that was challenging to bring back to solution, which likely led to considerable nucleic acid losses and renders this method unreliable for use with milk.

**TABLE 4:** Host DNA depletion kit characteristics

Abbreviation	Kit	Method	Catalog no.
NEB	NEBNext microbiome DNA enrichment	Methylated host DNA capture	E2612
Qia	QIAamp DNA microbiome	Host DNA degradation with Benzonase	51704
Molz	Molzym ultra-deep microbiome prep	Host DNA degradation with MolDNaseB	G-020-025
PMA^*a*^	Propidium monoazide	Covalent attachment to free DNA	40019

a Not a commercial kit but an in-house protocol combining selective lysis and exposure to PMA and light.

**TABLE 5:** Descriptive statistics of log numbers of copies per milliliter of milk for host depletion

Target	Mean no. of copies/ml of milk (SD), no. of expts^*a*^				
	No treatment	20 μM PMA	Molz	NEB	Qia
Total bacterial DNA	6.18 (0.23), 12	5.25 (0.09), 12*	4.42 (0.40), 8*	5.99 (0.07), 12^*b*^	5.04 (0.17), 12*
Bovine DNA	5.41 (0.23), 12	4.03 (0.28), 12*	2.32 (0.03), 8*	4.56 (0.08), 12*	3.55 (0.34), 12*

a Asterisks represent a significant difference from the values for the no-treatment control (Tukey-adjusted *P* < 0.0001).

b Bacterial copy numbers were significantly different from those of the no-treatment control (Tukey-adjusted *P *= 0.0532).

As all host DNA depletion methods also led to the depletion of bacterial DNA, we chose to use the calculated log difference between bovine and bacterial DNA copy numbers as key metrics for evaluating the different host DNA depletion methods. Untreated samples showed an average of 0.77-log-higher bacterial DNA copy numbers than bovine DNA copy numbers (Fig. 6C). By comparison, the log difference after different host depletion methods ranged from 1.22 log (PMA method) to 2.09 log (Molzym kit). If treated samples underwent untargeted sequencing, this decrease in bovine copies would translate into negligible differences in terms of relative abundances of bacterial reads versus bovine reads given the approximately thousandfold difference between the bovine genome size and the average bacterial genome sizes.

### Enzymatic selective lysis of inoculated milk followed by PMA treatment differentially affects detection of Gram-positive and Gram-negative bacteria through qPCR.

In a final attempt to optimize a host depletion protocol that would be applicable to raw milk, we tested a lysis protocol that included a mild protease, with the objective of more efficiently permeabilizing mammalian cells while keeping bacterial cells intact. Because we were aware of potential biases that could be introduced by adding an enzymatic lysis step prior to PMA treatment, we decided to inoculate the samples tested with Gram-positive and Gram-negative bacteria to address potential differential bacterial permeabilization by subtilisin that would result in DNA inactivation by PMA binding. We also investigated whether the light source would have an effect on the efficiency of PMA binding by processing samples in parallel and exposing PMA-treated sample duplicates to either a halogen light source or a commercial apparatus designed for use with PMA-treated samples (BLU-V; Qiagen). We prepared two biological replicates with two technical replicates for each comparison, from which duplicate qPCRs were done.

**FIG 7:**
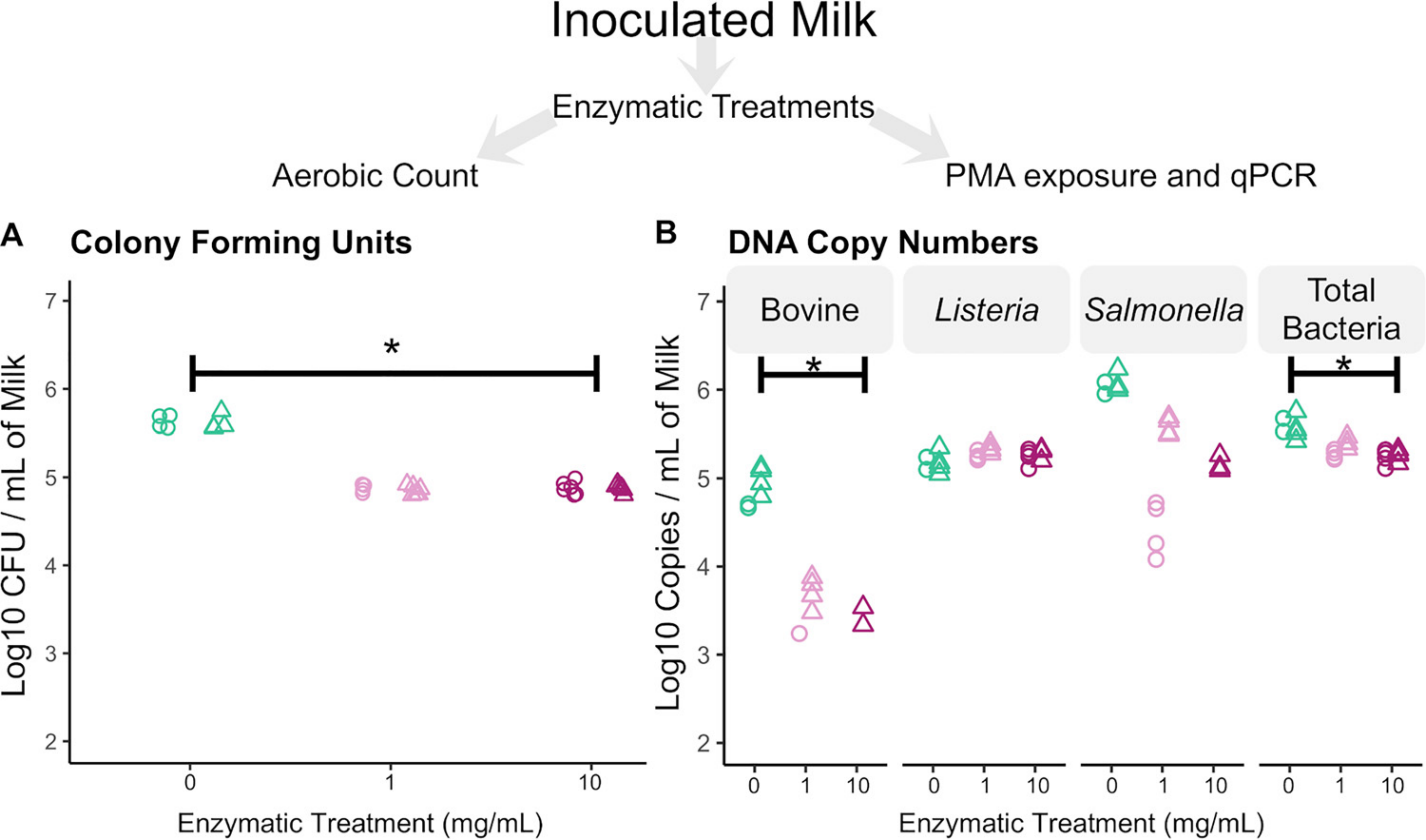
Comparison of enzymatic lysis results prior to host DNA depletion with 20 μM PMA in inoculated milk. (A and B) Aerobic standard plate count results (A) and qPCR results (B). Asterisks represent significant differences at a *P* of <0.05 (*) in linear-model comparisons. Different shapes correspond to two independent experiments with two technical replicates each, from which duplicate qPCRs were done, and colors correspond to the concentrations tested. CT values of samples in which melt curves did not match standard samples’ peaks were not used.

Treatment with subtilisin decreased the number of culturable bacteria as assessed through CFU plate counts of milk that had been treated with lysis solution prior to PMA exposure (Fig. 7A) (*P* < 0.001). However, we did not observe a difference in CFU counts in milk samples treated with different enzyme concentrations. The light source did not affect copy numbers (*P* = 0.74); therefore, qPCR comparisons were performed with combined data from both the halogen light source and BLU-V apparatus for Fig. 7B. As expected, we observed a decrease in bovine copy numbers in subtilisin-PMA-treated samples compared to those in the negative control that was much greater in extent than what we observed with osmotic lysis (Fig. 7B, first segment) (*P =* 0.04). However, we also observed a dose-dependent decrease in Gram-negative copy numbers in treated samples in the first replicate and were unable to detect Salmonella copy numbers at the higher enzyme concentration in the second replicate (Fig. 7B, third segment), while no differences were observed in Gram-positive copy numbers (Fig. 7B, second segment) (*P=* 0.3). This trend was confirmed by a less steep but still noticeable decrease in total bacterial copy numbers compared to the decrease in Gram-negative copy numbers (Fig. 7B, fourth segment) (*P=* 0.001). These data led us to conclude that enzymatic treatment followed by PMA inactivation differentially affects Gram-negative bacterial copy numbers, leading to biased results if used to prepare DNA for sequencing purposes.

## DISCUSSION

In this study, we comprehensively evaluated both nucleic acid extraction and host depletion protocols for bovine raw milk. The rationale of using raw milk as a model included the recent publication of several studies highlighting the variability of raw milk microbiotas (34–36), the potential influence of raw milk microbial quality on processed dairy products (37–39), and the fact that patterns of the milk microbiome represent potential “biomarkers” that can be tracked (20, 21). All of these studies highlight milk as a potential candidate for using HTS as part of quality assurance and risk assessment in the food industry in the future. Our results indicate that the following magnet-based DNA extraction methods are superior for extracting DNA from milk. Although host DNA depletion methods decrease the amount of bovine DNA in a given sample, the reduction is not sufficient for effectively depleting bovine DNA in HTS studies of raw milk. We also observed potential for bias introduction by certain protocols on the overall microbial profile, as well as selective bias against Gram-negative bacteria.

### Magnet-based DNA extraction seems to provide better results than column-based methods for raw milk samples.

We compared several nucleic acid extraction protocols used on bovine raw milk. While all protocols evaluated for extraction of DNA were able to successfully extract (albeit small amounts of) total DNA, we observed higher variability in replicates in some protocols than in others. The low DNA concentration from milk extracts is in agreement with those of previous reports (25, 26, 40). One particular aspect of this study is that we inoculated different types of bacteria that would be of interest in a dairy processing environment into raw milk and performed targeted qPCR to assess differential extraction of Gram-positive and Gram-negative bacteria. While most protocols were able to successfully extract DNA from the bacteria inoculated, magnet-based DNA extraction approaches had the best recovery and the lowest interreplicate variability, as evaluated by qPCR. It must be noted that the differences observed in the recovery of bacterial DNA are a characteristic of processing steps rather than a flaw or a sign of the inefficiency of a given protocol (i.e., while some extraction methods carry the entire sample lysate from one step to the following, some protocols require fixed or maximum volumes to be transferred to the next step due to volume limitations in the reaction tubes used). It is important to highlight that different DNA extraction methods have advantages and drawbacks, which can also vary according to the material to which they are applied. As no general gold standard method exists to date for this application, the selection of an extraction method should be based on the objectives of each particular study (41). Nevertheless, there is a growing consensus on the importance of mechanical disruption, such as bead beating for microbiome applications (42), and magnet-based extractions have been demonstrated to be particularly effective in the diagnosis of tuberculosis in the sera and plasma of patients (43) and in the extraction of algal DNA for next-generation sequencing (NGS) studies (44), highlighting magnetic methods as a common theme around diverse nucleic acid-based applications.

### Extracting bacterial RNA from high-quality raw milk is challenging.

The methods used here copurify both DNA and RNA or each nucleic acid separately, and based on the inoculation of bacterial loads in some cases exceeding regulatory standards for raw milk, we expected bacterial RNA to be detectable. We observed very low concentrations of extracted DNA and were unable to successfully detect bacterial RNA from our inoculated milk samples. This was a surprising finding, as literature on the milk metatranscriptome is available (19). However, the protocols previously used to successfully isolate RNA from milk involved extensive centrifugation of large sample volumes followed by several wash steps (45), as well as samples with much greater bacterial loads, including dairy products (such as cheese), which contain high levels of organisms responsible for fermentation of the products (19, 21).

### Current methods for host DNA depletion are not suitable for application in untargeted HTS studies of milk.

Because the bovine cells are present in milk along with the microbes of interest, and due to the striking difference between bovine and bacterial genome sizes, developing efficient yet unbiased host DNA depletion methods is critical for the adoption of HTS technologies in food safety. While we observed a decrease in the number of bovine copies compared to bacterial copies as assessed through qPCR in all protocols evaluated, this decrease is not sufficient to effectively change the relative abundance of reads being assigned to the bovine genome in HTS studies. This was confirmed when we performed deep untargeted sequencing of a subset of samples that had demonstrated promising results in qPCR but still detected over 99% of reads mapping to the bovine genome independent of host DNA depletion.

Among the other host DNA depletion protocols evaluated were immunoprecipitation of methylated eukaryotic DNA and selective lysis of mammalian cells followed by DNase treatment. One reason for the lack of efficiency observed in the treatment of milk with either of these methods may be the challenges associated with extracting DNA from milk in the first place. Such protocols require large amounts of good-quality DNA, which is practically impossible to obtain from milk samples without extensive centrifugation and pellet washes, potentially proving impractical in industry settings and representing added opportunities for sample contamination or unintentional bias of the microbiota for lipid- or protein-bound microorganisms. The characteristics of milk by themselves can also pose a challenge (presence of fats, proteins, and ions) (8, 9). Challenges specific to nucleic acid extraction from milk have been discussed in the literature to some extent, specifically by Metzger et al., who reported challenges in amplifying bacterial DNA from DNA extracted from milk (46).

To the best of our knowledge, this was the first study to evaluate host DNA depletion methods in a food matrix. The literature that tries to address host contamination in clinical sequencing applications for pathogen detection is limited, although improvements in the detection of malaria (47) and pathogens in infected tissue samples (48) and sputum (32) have been reported. Reports describing attempts to use host DNA depletion in cerebrospinal (30) and arthroplasty (29) fluids, as well as human saliva (28), are also available. The common theme around these studies was the various efficiencies across methods and sample types, highlighting the need for individual assessment of host depletion methods using the desired sample type.

### Enzyme-based selective lysis followed by PMA treatment differentially affects Gram-negative bacteria in inoculated raw milk.

Propidium monoazide has been extensively used to differentiate between live and dead bacterial cells due to its inability to penetrate intact cell membranes and has recently been indicated to be an effective method for host DNA depletion for use in HTS studies (28, 49, 50). We thus developed an enzyme-based method for host DNA depletion (which utilized a mild protease) combined with subsequent PMA treatment in an effort to better lyse mammalian cells while retaining intact bacterial cells.

While we detected an effect of the enzymatic treatment on total bacterial numbers as measured through plate counts that appeared to be independent of enzymatic concentration, we did not observe a decrease in Gram-positive bacterial numbers as measured by qPCR after PMA exposure. Nevertheless, in our spiking experiments, we observed an enzyme dose-dependent decrease of Gram-negative copy numbers in samples that underwent selective lysis followed by PMA exposure. These data led us to conclude that enzymatic treatment might have differentially permeabilized the membranes of Gram-negative cells, allowing PMA to bind to DNA without observable differences in overall bacterial survival. Taken together, these observations suggest that potential biases may occur against detection of Gram-negative bacteria through qPCR by this method.

### It is imperative to optimize protocols for samples with different characteristics.

The generalizability of this study lies in the importance of the standardization and validation of methods for each specific food matrix. It is also important to highlight the challenges associated with low bacterial biomass samples and the fact that various foods have a wide range of biomasses, from very low microbial content in raw milk to relatively high microbial content in some cultured dairy products. Efforts to standardize and recommend best practices in HTS studies, particularly pertaining to low-biomass samples (51–53), have recently begun and must be continued.

### Conclusions.

Our results suggest that magnet-based extraction methods are superior for bacterial nucleic acid isolation from bovine milk. Host DNA remains a challenge for untargeted sequencing of milk, highlighting that the food matrix characteristics should always be considered whenever planning HTS studies. Enzymatic-lysis-based PMA host depletion introduced dose-dependent biases against Gram-negative bacteria, suggesting that selective lysis permeabilized Gram-negative organisms to PMA, which subsequently hindered our ability to detect Gram-negative bacteria through qPCR without affecting counts of live bacteria. While it is not possible to test all methods available at any given time, we focused on kits and protocols that have been the most widely used for the extraction of nucleic acids from milk. As procedures are improved or new methods are developed, a reevaluation of available protocols would prove useful, as the development of HTS-based tools to aid and improve quality assurance and food safety programs continues to hold great promise.

## MATERIALS AND METHODS

### Comparison of nucleic acid extraction protocols in spiked milk. (i) Strain selection.

We selected a Gram-positive bacterium (Listeria monocytogenes), a Gram-negative bacterium (Salmonella enterica), and a sporeformer (Bacillus wiedmannii) previously isolated from milk or the dairy environment to create a mock microbial community that would be inoculated into raw milk. We included an additional Gram-positive bacterium, a member of the *Mycobacteriaceae* family (Mycobacterium smegmatis), because of the potential public health implications and the uniqueness of the cell structure of mycobacteria. Milk samples were specifically inoculated with Salmonella enterica, Listeria monocytogenes, and Mycobacterium smegmatis vegetative cells grown to stationary phase and Bacillus wiedmannii spores. Specific strain information is available in Table 6 and in the Food Microbe Tracker database (www.foodmicrobetracker.com). The bacterial load of the inoculated milk sample was chosen to represent the largest bacterial concentration allowed for raw milk in the United States, which is 300,000 CFU per ml, in an attempt to simulate the highest legal bacterial load of incoming milk in a dairy processing plant (54).

**TABLE 6:** Organisms used in this study

Target	Isolate	Source	NCBI accession no.^*a*^
Total bacterial DNA/RNA	Pooled culture		NC_000913
Bacillus wiedmannii	FSL H7-0344	Pasteurized 2% milk	NC_004722
Listeria monocytogenes	FSL A5-0145	Raw milk	NC_003210
Mycobacterium smegmatis	FSL X3-0054	VanDerVen lab (MC2155)	NC_008596
Salmonella enterica	FSL A5-0218	Raw milk	NC_003197
Bovine DNA	Bovine blood	Cornell University dairy farm	NM_001037471.2

a NCBI accession numbers of genomes used for primer design. Primers for the detection of the total bacterial DNA target 16S rRNA gene were designed based on conserved regions of the 16S rRNA gene.

### (ii) Inoculum preparation.

Bacillus wiedmannii spore suspension was prepared according to Buehler et al. (55). Briefly, the bacterial isolate was streaked from frozen culture into brain heart infusion (BHI) agar (Becton, Dickinson and Co., Sparks, MD) and incubated for 24 h at 37°C. Following incubation, a single colony was selected to inoculate a tube containing 5 ml of BHI broth, followed by incubation at 37°C for 72 h. Next, 100 μl of inoculated BHI was spread plated in duplicate on a sporulating medium, AK agar number 2 (Becton, Dickinson and Co.), which was incubated for 120 h at 37°C. Sporulation was confirmed via microscopy with a 7.5% malachite green endospore stain (JT Baker, Phillipsburg, NJ) as detailed in Gaillard et al. (56). Spores were harvested by flooding the agar surface with 10 ml of phosphate-buffered saline (PBS) (Weber Scientific, Hamilton, NJ) and scraping the bacterial culture with a cell scraper. Harvested cells were transferred to a sterile centrifuge tube and washed with 10 ml of sterile water three times by centrifugation at 10,500 rpm for 15 min and resuspension of the pellet. Following the third wash, 5 ml of sterile water and 5 ml of 100% ethanol (Decon Labs, King of Prussia, PA) were added to the tube, and the pellet was resuspended by vortexing. The bacterial pellet resuspended in 50% ethanol was incubated for 12 h at 4°C in a rotating platform to eliminate any remaining vegetative cells. After ethanol treatment, the spore suspension was washed another three times with 10 ml of sterile water as described above. The final spore suspension was kept in sterile water at 4°C until used for spiking experiments.

Mycobacterium smegmatis cells were streaked from frozen stocks into BHI agar, followed by incubation at 37°C for 48 h. A single colony was used to inoculate a 5-ml tube containing BHI broth with 1% Tween 80 (Fisher Scientific, Hampton, NH, USA), followed by incubation at 37°C for 48 h. The final inoculum was prepared by inoculating 100 μl of liquid culture onto 100 ml of prewarmed BHI broth with 1% Tween 80, followed by incubation at 37°C for 72 h. The resulting stationary-phase culture was kept at 4°C until it was used to spike milk samples.

Listeria monocytogenes and Salmonella enterica cells were streaked separately from frozen stocks into BHI agar, followed by incubation at 37°C for 24 h. For each strain, a single colony was used to inoculate a 5-ml tube containing BHI broth, followed by incubation at 37°C for 24 h. The final inoculums were prepared by inoculating 100 μl of each liquid culture onto 100 ml of prewarmed BHI broth, which was subsequently incubated at 37°C for 12 h.

At harvesting, bacteria were spiral plated on agar using an Eddy Jet 2W spiral plater (IUL Micro, Barcelona, Spain) at various dilutions to determine bacterial concentrations. Bacterial liquid cultures were kept at 4°C until bacterial enumeration (B. wiedmannii, L. monocytogenes, and Salmonella sp. were kept for 24 h, and M. smegmatis was kept for 48 h at 4°C). Inoculum volumes were calculated based on CFU.

### (iii) Inoculation of raw milk.

Raw milk was collected from the Cornell University Ruminant Center (CURC; Hartford, NY) bulk tank into sterile 10-oz lock tab containers (Capitol Plastics, Amsterdam, NY) and transported on ice to the Milk Quality Improvement Program laboratory in Ithaca, NY. Samples were combined into a single sterile 1,000-ml glass bottle and homogenized by inverting the container 50 times. From that bottle, one aliquot (1 ml) was used to make dilutions and determine the initial bacterial count using a standard plate count (SPC) agar (Millipore Sigma, Burlington, MA), which was incubated for 48 h at 32°C. Bacterial enumeration was performed using an automated colony counter (SphereFlash automatic colony counter, IUL Micro, Barcelona, Spain). A second aliquot (60 ml) was transported on ice to the DairyOne laboratory (DairyOne, Ithaca, NY) for determination of somatic cell counts (SCC) and milk contents (fat, protein, lactose, and total solids). The remaining milk was used for inoculation with the strains described above. To allow for interaction between inoculated bacteria and milk components and to mimic conditions similar to those when raw milk is stored in dairy silos, inoculated milk was held at 4°C for 24 h prior to use as starting samples for nucleic acid extraction comparisons.

### (iv) Bacterial enumeration of inoculated milk samples.

Total bacterial enumeration was performed through serial dilutions of each milk sample in PBS, which was spiral plated in duplicate on standard plate count agar as described above and incubated for 48 h at 32°C.

### (v) Nucleic acid extraction.

Raw (uninoculated) milk samples were processed in parallel with inoculated milk samples for all kits. Additionally, a no-template nucleic acid extraction was carried out as a negative control to assess cross-contamination and potential reagent contamination. As a positive control, a mock bacterial community was created in PBS with the same bacteria described above at the same concentration as the inoculated milk samples; this was included on each extraction plate or run as a control. Inoculated milk samples were extracted in three technical replicates in each of three independent biological replicates, which were performed on separate days with a different raw milk sample used for each biological replicate.

Kits, manufacturers, and protocol details are described in Table 2. For each of the nine protocols evaluated, samples were processed in parallel (uninoculated milk, inoculated milk triplicates, mock bacterial community in PBS, and a negative control without a starting sample [“kit buffers only”]) according to the manufacturer’s instructions. Exact versions of protocols followed can be found in a GitHub repository as supplemental material (https://github.com/ErikaGanda/MilkDNA). Extracted nucleic acids were frozen at −80°C until quantification and qPCR assays were performed.

### (vi) Nucleic acid quantification.

Nucleic acid quantification was performed with both a spectrophotometer (Nanodrop 2000; ThermoFisher Scientific) and a fluorescence-based method. Absorbance was measured at 280, 260, and 230 nm for DNA and RNA. Total DNA was also measured with a Quant-iT double-stranded DNA (dsDNA) high-sensitivity (HS) assay kit (ThermoFisher Scientific); fluorescence measurements were performed using a Synergy H1 plate reader (BioTek Instruments, Winooski, VT, USA) with wavelengths of 490 nm for excitation and 535 nm for emission.

### (vii) qPCR primer development and assay conditions.

For each bacterial strain used to inoculate milk samples, qPCR primers were designed to target the RNA polymerase subunit beta gene (*rpoB*) because it is a single-copy gene and allows for a more accurate comparison between bacterial numbers than 16S rRNA genes. Primers were also designed to target a conserved region of the 16S rRNA gene, and calculated 16S copy numbers were used as a proxy for total bacterial numbers. Primer details are described in Table 7. Reactions were carried out in duplicate using 2 μl of extracted DNA. The final qPCR volumes totaled 25 μl and contained 12.5 μl SYBR green master mix (ThermoFisher Scientific), 2 μl extracted DNA, 0.5 μM forward primer, 0.5 μM reverse primer, and 9.5 μl nuclease-free water. Reactions were carried out in a QuantStudio 6 instrument (ThermoFisher Scientific), with the following cycling conditions: 95°C for 10 min followed by 40 cycles of 95°C for 15 s and 60°C for 1 min and a melting curve of 95°C for 15 s, 60°C for 1 min, and 95°C for 15 s.

**TABLE 7:** Primers used in this study

Target	Primer(s)	Sequence(s)	Fragment size (bp)	Source
Total bacterial DNA/RNA	EKG43-16-TotalBact16S-F2	GTAGCGGTAAATGCGTAGA	120	This study
	EKG43-4-TotalBact16S-R	GACTACCAGGGTATCTAATC		This study
Bacillus wiedmannii	JK2739-3rpoB_F	AACGTGCTTGTTGGCTTCAT	152	This study
	JK2739-4rpoB_R	TCTTCTGGTCCAAGCTTCGT		This study
Listeria monocytogenes	VGO-23-rpoB-RT-F	TCGTCGTCTTCGTTCTGTTG	221	Liu et al. (62)
	VGO-24-rpoB-RT-R	GTTCGCCAAGTGGATTTGTT		Liu et al. (62)
Mycobacterium smegmatis	EKG43-15-Mycobacterium-rpoB-F2	TCGGTGAGCTGATCCAGAAC	156	This study
	EKG43-14-Mycobacterium-rpoB-R	TGCCGAAGAACTCCTTGATC		This study
Salmonella sp.	EKG43-7-Salmonella-rpoB-F	GTACCGTCGTGTGGTTGATG	170	This study
	EKG43-8-Salmonella-rpoB-R	GGCTGAACAAGCTGGATTCG		This study
Bovine RNA	Bt03229278_m1	Ubiquitously expressed bovine transcript	89	Commercial assay
Bovine DNA	Bt03229276_g1	Ubiquitously expressed bovine transcript	88	Commercial assay

Bovine genome copy numbers were calculated using a commercial TaqMan assay targeting the UXT gene (ThermoFisher Scientific). The final qPCR totaled 20 μl and included 1 μl of the gene expression assay mixture, 10 μl TaqMan Fast advanced master mix (ThermoFisher Scientific), 7 μl of nuclease-free water, and 2 μl of the template. Reactions were carried out in a QuantStudio 6 instrument, with the following cycling conditions: 95°C for 20 s, followed by 40 cycles of 95°C for 1 s and 60°C for 20 s. For each of the three biological replicates, samples extracted with all kits were amplified in a single PCR plate and compared using the same standard curve.

### (viii) qPCR data analysis.

Amplification data were exported from QuantStudio real-time PCR software (ThermoFisher Scientific) into Excel (version 16.0.11325.20156; Microsoft Corp., Redmond, WA). Standard curves were built with serial dilutions of purified bacterial DNA that was quantified using a Nanodrop spectrophotometer (ThermoFisher), and genome equivalents in each reaction mixture were calculated as described in Brankatschk et al. (57). Standard curves with an *R*^2^ of <0.9 and an efficiency of <70% were discarded, and reactions were repeated. For 2 out of 24 reaction plates, one outlier was removed based on visual inspection of deviating standard curve data points.

Copies per microliter of the DNA input were calculated for each reaction. Melt curves were visually inspected, and cycle threshold (CT) values of samples in which melt curves did not match standard samples’ peaks were not used for final copy number calculations. Because different kits required various amounts of sample input and nucleic acid output (Table 2), data were normalized to allow for comparison between kit protocols. We chose to simulate copy numbers in 1 ml of milk input and 100 μl DNA output for each kit, using the following equations:

$$\text{milk equivalents}=\frac{\text{input milk sample }(µ\text{l})}{\text{eluted DNA (}µ\text{l)}}$$

$$\text{copies/ml of milk}=\frac{\text{copies/}µ\text{l of DNA }\times \text{ 1,000}}{\text{milk equivalents}}$$

Normalized copy number data were log transformed prior to statistical analysis. The final data set was cross-referenced and checked with the open-source software OpenRefine (https://github.com/OpenRefine). All statistical analyses with inoculated milk PCR data were performed in R (version 3.4.3; R Project, Vienna, Austria).

### (ix) Statistical analysis.

To describe the differences observed between extraction methods, linear models were fit using the lm function in R comparing log copy numbers between kits. A separate linear model was fit for each assay, and all linear models included kit/method, biological replicate (SpikeSet), and their interactions. Two observations (one from PowerFood in the Bacillus wiedmannii assay and one from CORE in the total bacterial DNA assay) had Cook’s distance greater than 0.5, were flagged as outliers, and were removed from subsequent analyses. The linear model was then refitted excluding these two observations. For each assay, a two-tailed pairwise comparison of means was performed using the R package multcomp (58). Two kits were removed from these comparisons due to identifiability issues stemming from too few nonmissing observations (too many failed reactions). E.Z.N.A. Food DNA had failed reactions across all assays except the bovine assay, and PowerViral had failed reactions in all of the first biological replicates of the *Listeria* assay. All *P* values (combined across the 6 assays) were corrected to achieve an overall family-wise error rate less than or equal to 0.05 using the Bonferroni correction (59). Raw data and code are available in a GitHub repository (https://github.com/ErikaGanda/MilkDNA).

### Host depletion protocols. (i) Selective osmotic lysis of host cells and host DNA depletion through PMA treatment of uninoculated raw milk.

An osmotic lysis-based host DNA depletion protocol was adapted from Marotz et al. (28). Briefly, 500 μl of uninoculated milk were centrifuged at 10,000g for 8 min, whey was discarded while fat and pellet were kept in the microcentrifuge tube. Five hundred microliters of sterile double distilled water (ddH_2_O) were added to the pellet. The tube was vortexed until pellet dissolution, followed by incubation at room temperature for 5 min to allow for osmotic lysis of mammalian cells. We compared four different concentrations of propidium monoazide (PMA; catalog number 40019; Biotium, Hayward, CA): 10, 20, 40, and 50 μM. Untreated milk (milk that was not centrifuged or exposed to osmotic lysis) and milk exposed to osmotic lysis but not exposed to PMA were also included in comparisons. To account for biological variation and assess repeatability, experiments were performed in three biological replicates performed on three different days, using a different raw milk sample for each biological replicate.

After incubation at room temperature, the appropriate volume of PMA was added to each tube to achieve desired concentrations, followed by a brief mixing and incubation in the dark (in an aluminum foil-wrapped box) for 5 min on a rotating platform.

Following PMA incubation in the dark, PMA was inactivated by light exposure. Samples were placed horizontally on ice <20 cm from a 500-W halogen light source (Woods halogen work light; Southwire Company LLC, Carrollton, GA, USA). Samples were exposed to light on a rotating platform for 5 min and frozen at −80°C until DNA extraction with a magnet-based method (MagMAX CORE nucleic acid purification kit; ThermoFisher). Quantitative PCRs and data analysis were performed as described above, with the exception that only bovine DNA and total bacterial copy numbers were quantified, as no bacteria were inoculated in the milk samples used. Linear models were fitted to assess the effect of selective lysis in non-PMA-treated samples (UTC versus treatment with 0 μM) and the effect of PMA concentration on copy numbers.

### (ii) Selective enzymatic lysis of host cells and host DNA depletion through PMA treatment of inoculated raw milk.

Because milk has more fat, protein, and minerals than saliva, we hypothesized that the 1:1 osmotic lysis included in the protocol adapted from Marotz et al. (28) was not optimal for lysing bovine cells in milk compared to that for lysing human cells in saliva.

We thus also evaluated a combination of a mild lysis solution followed by incubation with two concentrations of subtilisin, a protease from Bacillus licheniformis (Krackeler Scientific; catalog number 45-P5380-25MG). The lysis solution contained 7.6 g/liter sodium carbonate, 8.8 g/liter sodium bicarbonate, 2.43 g/liter disodium EDTA, and 2.71 g/liter tetrasodium EDTA. Reagents were solubilized in 1 liter of sterile double-distilled water using a stir plate with a magnetic rotating bar for 6 h, prior to pH measurement, titration to 9.5, and filter sterilization with a 0.22-μm filter (Corning disposable vacuum filter; Fisher Scientific, Waltham, MA).

Experiments were performed on two separate days with a newly collected milk sample on each day. To access potential lysis biases, we spiked milk with a Gram-positive and a Gram-negative bacterium as described above (L. monocytogenes and Salmonella sp.). Spiked milk samples were treated prior to DNA extraction as follows. Four hundred microliters of inoculated milk was added to 1.6 ml of lysis solution and incubated at room temperature for 5 min prior to the addition of subtilisin. Two concentrations of subtilisin were tested: 10 mg/ml (10×) and 1 mg/ml (1×) to achieve final concentrations of 0.73 and 0.073 units per reaction mixture, respectively. After addition of the enzyme, samples were incubated at 50°C for 5 min and placed on ice after incubation. Samples were then centrifuged at 10,000 × *g* for 8 min. The supernatant-containing lysis solution and enzyme were discarded, and the pellet was resuspended with 400 μl of PBS. Treated samples were exposed to PMA at 20 μM as described above, photoactivated using two light sources (the halogen lamp described above or the BLU-V system from Qiagen) and stored at −80°C until DNA extraction with a magnet-based method (MagMAX CORE extraction kit; ThermoFisher).

Quantitative PCRs and data analysis were performed as described above, with the exception that bovine DNA, total bacterial copy numbers, L. monocytogenes cells, and Salmonella cells were quantified, as we hypothesized that the lysis solution could affect Gram-negative bacteria differently than Gram-positive bacteria.

### (iii) Comparison of host DNA depletion methods in uninoculated raw milk.

Based on initial experiments with various PMA concentrations we decided to include 20 μM in a comparison with three commercial host DNA depletion kits. Kits, manufacturers, and protocol details are described in Table 4. Samples were processed according to the manufacturer’s instructions and frozen at −80°C.

For the NEB host depletion method, DNA was extracted with a magnet-based extraction procedure and quantified using a qubit fluorometer (ThermoFisher) prior to methylated host DNA capture reactions. Final microbially enriched DNA cleanup was performed using AMPure magnetic beads (Beckman Coulter). Exact versions of protocols followed can be found in our GitHub repository (https://github.com/ErikaGanda/MilkDNA). Three biological replicates (each with two technical replicates) were prepared using a new sample of raw milk for each biological replicate (except with the Molzym kit, which was performed only with duplicate milk samples in 1 day due to limited available reagent amounts).

Quantitative PCRs and data analysis were performed as described above, with the exception that only bovine DNA and total bacterial copy numbers were quantified, as no bacterium was inoculated in the milk samples used.

### (iv) Sequencing.

In addition to qPCR, we performed deep untargeted sequencing of a subset of nine samples at the Cornell Biotechnology Resource Center. Briefly, DNA quality control was performed in a fragment analyzer, and sequencing libraries were constructed using a Nextera DNA flex kit (now renamed Illumina DNA Prep; Illumina, San Diego, CA) and 2× 150-bp paired-end sequencing was performed in an Illumina NextSeq500. Sequencing data quality control and *in silico* host signal removal were performed as previously described (3, 4), and microbial data were processed as described in the work of Beck et al. (4). Briefly, adapter removal and quality trimming were performed with TrimGalore (60), and trimmed reads of at least 50 bp in length were classified using Kraken v0.3 (61) against a multieukaryote database of 31 common food ingredients and contaminants, including the bovine reference genome (GCF_000003205.7, Btau_5.0.1) for bioinformatic removal of the host signal. Then taxonomic profiling of samples was completed using Kraken v0.3 against microbial RefSeq genomes as described by Beck et al. (4). A study diagram is presented in Fig. 1.

### Data availability.

The data sets and R code are available in our GitHub repository: https://github.com/ErikaGanda/MilkDNA. Raw sequencing reads have been deposited for all 9 sequenced samples into SRA under the BioProject accession number PRJNA667736.

10.1128/mSystems.00619-21.3 FIG S1 Detailed qPCR results, including for the no-template control. Download FIG S1, TIF file, 13.3 MB. Copyright © 2021 Ganda et al. 2021 Ganda et al. https://creativecommons.org/licenses/by/4.0/ This content is distributed under the terms of the Creative Commons Attribution 4.0 International license.
