# Supplementary figures and images for: DNA Extraction and Host Depletion Methods Significantly Impact and Potentially Bias Bacterial Detection in a Biological Fluid
Source: mSystems. 2021 Jun 15;6(3):e00619-21. doi: 10.1128/mSystems.00619-21 (PMC8574158; doi:10.1128/mSystems.00619-21)

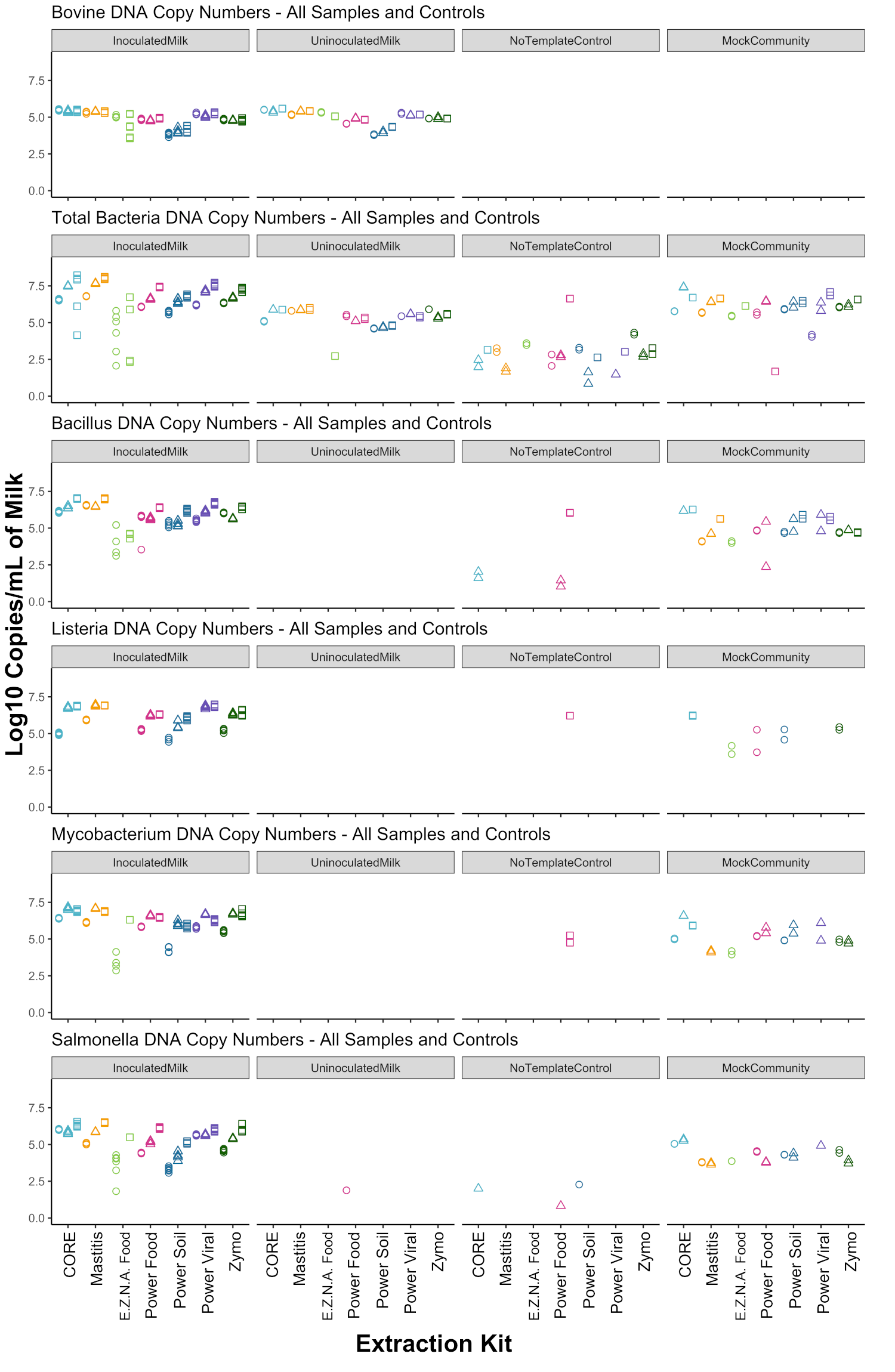

Supplement: FIG S1 [file msystems.00619-21-sf001.tif]
